# Impact of Obesity on Outcomes of Operable Breast Cancer: A Retrospective Cohort Study

**DOI:** 10.31557/APJCP.2020.21.4.953

**Published:** 2020-04

**Authors:** Tanapat Engkakul, Nuntakorn Thongtang, Akarin Nimmannit, Suebwong Chuthapisith, Charuwan Akewanlop

**Affiliations:** 1 *Division of Medical Oncology, *; 2 *Division of Endocrinology and Metabolism, Department of Medicine, *; 4 *Division of Head, Neck and Breast Surgery, Department of Surgery, Faculty of Medicine, *; 3 *Office for Research and Development, Siriraj Hospital, Mahidol University, Bangkok, Thailand*

**Keywords:** Breast cancer, obesity, body mass index, disease, free survival, prognosis

## Abstract

**Objective:**

Obesity is increasing worldwide. Previous studies of the impact of obesity on breast cancer outcomes have reported conflicting results. We investigated the association of obesity and breast cancer survival in Thai patients.

**Methods:**

Medical records of operable breast cancer patients diagnosed and treated at Siriraj Hospital between January 2004 and December 2011 were reviewed. Demographic data, tumor characteristics, stage, treatment and adverse event were described. Obesity was defined as body mass index (BMI) ≥ 25 kg/m^2^ using Asian’s cutoff value. Survivals in both obese and non-obese patient groups were analyzed.

**Results:**

A total of 400 patients were included, 200 in each group. Obese patients were older and associated with more comorbidity. Obesity was associated with larger tumor size (p = 0.011), greater numbers of lymph node involvement (p = 0.003) and more advanced stage (p = 0.01). Obese patients were more likely to receive less adjuvant chemotherapy and hormonal treatment. There was no statistically significant difference in disease-free survival (DFS) (Hazard ratio [HR] 0.72, 95% confidence interval [CI] 0.46 to 1.13) and overall survival (OS) (HR 0.77, 95% CI 0.43 to 1.39) between obese and non-obese patients. Interestingly, obesity was associated with fewer complications from chemotherapy than non-obese patients (p = 0.047).

**Conclusion:**

Obesity had no adverse prognostic impact association on both DFS and OS in Thai patients with operable breast cancer, although obese patients more often presented with larger tumor and higher numbers of lymph node involvement.

## Introduction

Body mass index (BMI) defined as weight in kilograms divided by height in meters squared (kg/m^2^) generally correlates highly with adiposity. BMI is easy to calculate and has been recommended as the measure of obesity in adults in all studies (WHO/IASO/IOTF, 2000). According to WHO classification, obesity is defined by BMI ≥ 30 kg/m^2^ in adult Europeans, whereas in adult Asians, obesity refers to BMI ≥ 25 kg/m^2^ (WHO/IASO/IOTF, 2000; Anuurad et al., 2003; WHO Expert Consultation, 2004). The prevalence of obesity has been rising every year (Smith and Smith, 2016). Obesity becomes global concern and is associated with increasing risk of cardiovascular disease, metabolic syndrome and various cancers (Goodwin and Stambolic, 2015; Berrigan et al., 2016; Global BMI Mortality Collaboration, 2016). The explanation of these emerging events has been described in few researches as obesity increases numbers of pre-adipocytes and inflammatory cells, higher levels of leptin and free fatty acids, and greater release of cytokines and other inflammatory compounds (Goodwin, 2015; Orecchioni et al., 2015).

Breast cancer is the most frequently diagnosed cancer and the leading cause of cancer death among females worldwide (Torre et al., 2012). Obesity increases risk of breast cancer (Goodwin, 2015). Several studies demonstrated that breast cancer patients with obesity have worse outcome with regard to disease free survival (DFS) and overall survival (OS) than those who were none-obese (de Azambuja et al., 2010; Conroy et al., 2011; Widschwendter et al., 2015; Kawai et al., 2016). Weight gain after diagnosis of breast cancer is associated with higher all-cause mortality rates compared with maintaining body weight (Playdon et al., 2015). In contrast, some studies reported that obesity has no impact on breast cancer prognosis in node-positive breast cancer patients receiving modern adjuvant chemotherapy (Pajares et al., 2013; Ladoire et al., 2014). However, most of these studies presented data from Western countries where obesity defined as a BMI ≥ 30 kg/m^2^ which is different from Asian population. To the best of our knowledge, the impact of obesity on breast cancer prognosis in Thai patients has never been reported. We decided to conduct an analytical-retrospective cohort study to answer this question.

The objective of the present study was to compare survival outcome between obese and non-obese patients with operable breast cancer, defining obesity as a BMI ≥ 25 kg/m^2^. In addition, we performed analysis to evaluate the association between BMI and clinical characteristics of breast cancer patients as well as toxicity related to systemic treatment. 

## Materials and Methods

This retrospective cohort study was conducted by reviewing the medical records of patients with stage I, II and III (pT1-4, pN0-3 and M0) according to the 7^th^ edition of the American Joint Committee on Cancer (AJCC) cancer staging (Edge and Compton, 2010). Patients were diagnosed and treated at Siriraj Hospital between January 1, 2004 and December 31, 2011. Patients’ medical records were selected by using ICD-10 coding from the hospital database. The selection started from the most recent record to the older ones until the calculated sample size was obtained. We excluded patients noted to have edema, serum albumin less than 3.0 g/dL and those who did not complete treatment or were lost to follow-up. The study was approved by Siriraj Institutional Review Board, Faculty of Medicine Siriraj Hospital, Mahidol University, Thailand (Protocol number 750/2559(EC1)).

Demographic data (age, weight at diagnosis, height, comorbidity, menopausal status, family history of breast cancer, history of hormonal use and pregnancy), date of diagnosis, tumor characteristics, TNM stage, treatment (surgery, radiation therapy and systemic therapy), adverse event, date of disease recurrence or death, date of last follow-up and weight at recurrence or last follow-up were collected. BMI was calculated as weight in kilograms divided by the square of height in meters. Patients were classified as non-obese (BMI < 25 kg/m^2^) and obese (BMI ≥ 25 kg/m^2^) group. Definition of disease-free survival (DFS) is the interval between the date of diagnosis and the date of disease recurrence or death; overall survival (OS) is the interval between the date of diagnosis and the date of death from any cause. Hormone receptor and HER2 status were determined according to American Society of Clinical Oncology / College of American Pathologists guideline recommendations for testing of estrogen and progesterone receptors in 2010 (Hammond et al., 2010) and for HER2 testing in 2013 (Wolff et al., 2013), respectively.

The primary objective was to determine the association of obesity on DFS; the secondary objectives were to evaluate the impact of obesity on OS, the association of BMI with patient and tumor characteristics as well as toxicity related to systemic treatment using Common Terminology Criteria for Adverse Events (CTCAE) v.4.0. 

*Statistical analysis*

This study was aimed to determine whether there is a statistically significant difference in 5-year DFS between non-obese and obese breast cancer patients. Based on Widschwendter (2015), estimated 5-year DFS in the non-obese group was 90%. A decrease from 90% to 80% 5-year DFS in the obese group was considered to be clinically relevant. Assuming a two-sided, type I error of 0.05 with at least 80% power, a study sample of 200 patients in each group was required (total number of patients was 400). 

Patient characteristics and outcomes were described using descriptive statistics, including frequency and percentage for categorical variables. Continuous variables were reported as mean, standard deviation of normally distributed variables and median, minimum and maximum where appropriate. For between group comparisons, we used Pearson’s chi-squared test or the Fisher exact test for categorical variables and Student’s t-test or Mann-Whitney U test for continuous variables. The Kaplan-Meier method of survival analysis was used to estimate survival and comparison between groups by log rank test. The Cox proportional hazards regression was used to estimate the hazard ratio (HR) and 95% CI for univariate and multivariate analysis of DFS and OS. For all tests performed, a two-tailed p-value < 0.05 was considered statistically significant. All analyses were performed using PASW Statistics (formerly SPSS Statistics) version 18.0 (SPSS, Inc., Chicago, IL).

## Results

A total of 511 patients’ medical records were reviewed to obtain the calculated sample size. Fifty-one patients were stage IV at diagnosis. Twenty-five and 35 patients were excluded due to referral to other hospitals for reimbursement issue and loss to follow-up, respectively. 

*Association of BMI with patient and tumor characteristics*

There were 200 operable breast cancer patients in each group (obese and non-obese). All were females with a mean age of 52 ± 11.1 years. Non-obese patients were significantly younger (mean age 50.1 ± 10.9 years) and tended to be premenopausal (57.5%) compared with obese patients (mean age 53.9 ± 11.0 years; 48% were premenopausal). Obese patients had significantly higher number of comorbidities such as type 2 diabetes mellitus (DM) and hypertension when compared with non-obese. Obesity was significantly associated with larger primary tumor size, increased numbers of lymph node involvement and more advanced tumor stage (Table 1). We observed no significant differences in tumor grade, hormone receptor and HER2 status between obese and non-obese patients. 

With regard to treatment received, there was no difference in type of surgery between the two groups. However, obese patients were more likely to receive less chemotherapy (83.5% vs. 92.5%, p = 0.006) and hormonal treatment (72% vs. 80.5%, p = 0.046) when compared with non-obese patients (Table 1). There was no significant difference in the chemotherapy regimen received in obese and non-obese patient groups. The majority of patients (94%) received anthracycline and taxane-based regimens. (Table included in supplement data).

*Effect of BMI on disease-free and overall survival*

**Figure 1:**
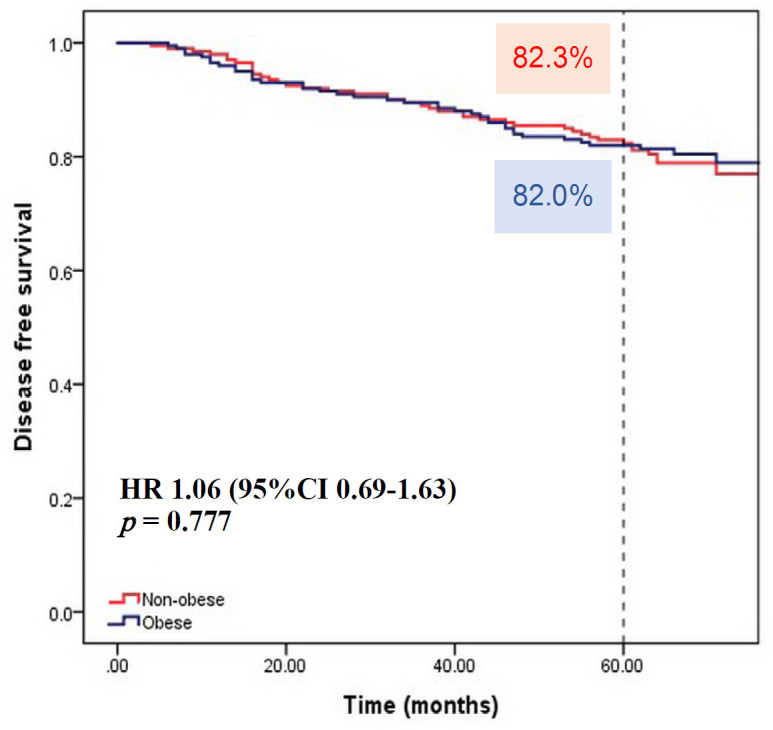
Kaplan Meier Survival Curve of Disease-free Survival According to BMI group (Non-obese, BMI < 25 kg/m^2^; Obese, BMI ≥ 25 kg/m^2^)

**Figure 2:**
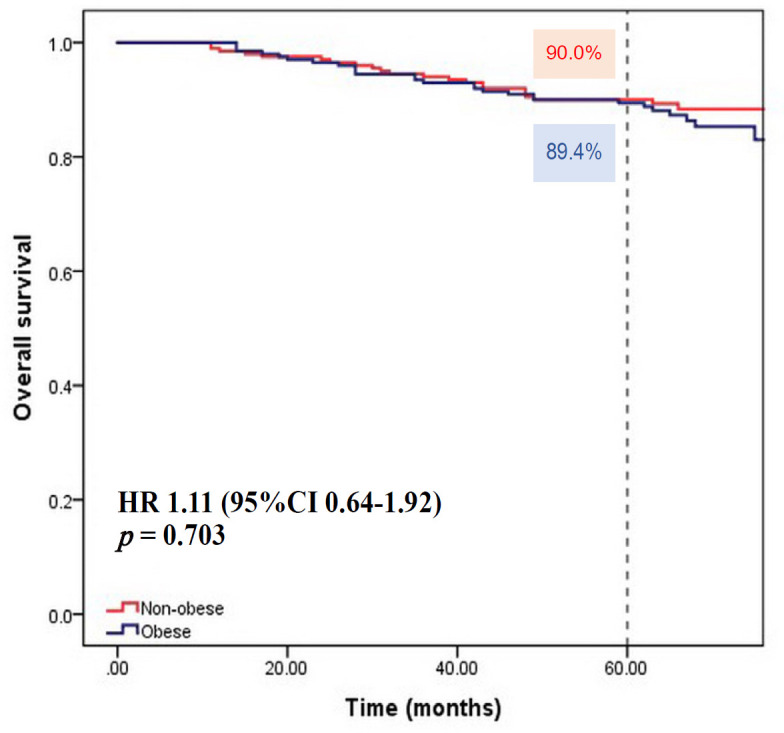
Kaplan Meier Survival Curve of Overall Survival According to BMI group. (Non-obese, BMI < 25 kg/m^2^, Obese, BMI ≥ 25 kg/m^2^)

The median follow-up time of 400 patients was 65 months (range 11-136 months). At the last date of follow-up (February 28, 2017), there were 347 patients alive, 52 patients deceased, 1 patient lost to follow-up. Of the 200 patients in each group, 41 and 43 patients in the obese and non-obese group had disease recurrence, respectively. There was no statistically significant difference in 5-year DFS between the two group, 82.3 % for the non-obese and 82% for the obese group (Hazard ratio [HR] 0.94; 95% confidence interval [CI] 0.61 to 1.44, p = 0.779) (Figure 1). In term of OS, there was no statistically significant difference between the non-obese and obese patients, with 5-year OS of 90% and 89.4%, respectively (HR 1.11; 95% CI 0.64 to 1.92, p = 0.703) (Figure 2). 

Since there was no significant difference in survival using cut-off of BMI ≥ 25 kg/m^2^, we then performed analysis using the cut-off of BMI ≥ 30 kg/m^2^ for obesity as in European population, 5-year DFS was 82.9% in the non-obese patients and 77.3% in the obese group. The difference was not statistically significant either (HR 1.35; 95% CI 0.76 to 2.39, p = 0.307). Five-year OS was 90.2% and 86.6% in the group with BMI < 30 kg/m^2^ and BMI ≥ 30 kg/m^2^ respectively (HR 1.68; 95% CI 0.84 to 3.37, p = 0.14).

Univariate analysis was performed using clinical parameters and known prognostic factors to evaluate for the significant influence on DFS and OS. These factors included age, comorbidity, BMI, tumor size, lymph node involvement, hormone receptor status, HER2 status and treatment with chemotherapy. Large tumor (T3 and T4), hormone receptor negative and HER2 positive were associated with worse DFS and OS (Table 2). 

Multivariate Cox proportional hazard regression analysis of factors affecting DFS and OS was performed using factors mentioned previously. Independent risk factors for worse DFS were having comorbidity, T3 and T4 tumor and hormone receptor negative. Factors that remained significant for poorer OS were large T3 and T4 tumor, hormone receptor negative and HER2 overexpression (Table 2). BMI did not have prognostic effect on both DFS and OS.

*Complication of systemic treatment*

Of the 352 patients receiving chemotherapy, obese patients experienced significantly less toxicity from chemotherapy with a lower rate of grade 4 neutropenia and febrile neutropenia when compared with non-obese patients (p <0.001 and p = 0.038, respectively). Moreover, non-obese tended to require more dose reduction in subsequent cycles when compared with obese patients. There was no significant difference in side effects from hormonal treatment between the two groups (Table 3).

**Table 1:** Baseline Patient Characteristics

	BMI < 25 mg/m^2^ N=200	BMI ≥ 25 mg/m^2^ N=200	*P*-value
Age (years)			
Mean (SD)	50.1 (10.9)	53.9 (11.0)	0.001*
Range	21 - 80	27 - 81	
BMI (mg/m^2^)			
Mean (SD)	21.7 (1.9)	28.6 (3.3)	<0.001*
Range	15.4– 24.9	25.0– 40.9	
Menopausal status- no. (%)			
Premenopausal	115 (57.5)	96 (48)	0.057
Postmenopausal	85 (42.5)	114 (52)	
Comorbidities- no. (%)			
No	148 (74)	108 (54)	<0.001*
Yes	52 (26)	92 (46)	
Type 2 DM	10	35	<0.001*
Hypertension	29	72	<0.001*
Dyslipidemia	25	30	0.48
Tumor size - no. (%)			
T1	45 (22.5)	31 (15.5)	0.011*
T2	124 (62)	118 (59)	
T3	12 (6)	24 (12)	
T4	19 (9.5)	25 (12.5)	
Unknown	0 (0)	2 (1)	
Nodal stage - no. (%)			
N0	70 (35)	59 (29.5)	0.003*
N1	83 (41.5)	64 (32)	
N2	31 (15.5)	38 (19)	
N3	16 (8)	39 (19.5)	
Histological grade - no. (%)			
G1	18 (9)	14 (7)	0.5
G2	109 (54.5)	124 (62)	
G3	59 (29.5)	50 (25)	
Unknown	14 (7)	12 (6)	
Stage - no. (%)			
IA	4 (2)	2 (1)	0.01*
IIA	94 (47)	69 (34.5)	
IIB	40 (20)	40 (20)	
IIIA	30 (15)	36 (18)	
IIIB	16 (8)	17 (8.5)	
IIIC	16 (8)	36 (18)	
Histological type -No. (%)			
Invasive ductal	180 (90)	185 (92.5)	0.571
Invasive lobular	10 (5)	9 (4.5)	
Other	10 (5)	6 (3)	
Hormone receptor status- no. (%)			
Negative	38 (19)	53 (26.5)	0.074
Positive	162 (81)	147 (77)	
HER2 status - no. (%)			
Negative	140 (70)	130 (65)	0.408
Positive	48 (24)	52 (26)	
Unknown	12 (6)	18 (9)	
Type of surgery - no. (%)			
BCS	50 (25)	40 (20)	0.253
Mastectomy	150 (75)	158 (79)	
Chemotherapy - no. (%)			
No	15 (7.5)	33 (16.5)	0.006*
Yes	185 (92.5)	167 (83.5)	
Neoadjuvant			
No	174 (87)	166 (83)	0.254
Yes	26 (13)	34 (17)	
Adjuvant			
No	27 (13.5)	42 (21)	0.047*
Yes	173 (86.5)	158 (82.8)	
Hormonal treatment no. (%)			
No	39 (19.5)	56 (28)	0.046*
Yes	161 (80.5)	144 (72)	
AntiHER2 - no. (%)			
No	180 (90)	180 (90)	1
Yes	20 (10)	20 (10)	

BCS, Breast conserving surgery; *p-value < 0.05 statistical significance

**Table 2:** Univariate and Multivariate Analyses of Factors Affecting Disease-free Survival (DFS) and Overall Survival (OS)

DFS	Univariate analysis (N = 400)			Multivariate analysis (N = 400)		
	HR†	95% CI‡	*P*-value	HR†	95% CI‡	*P*-value
BMI (kg/m^2^)						
BMI < 25	1			1		
BMI ≥ 25	1.06	0.69-1.63	0.777	0.72	0.46-1.13	0.159
Age						
≥ 50	1			1		
< 50	1.42	0.92-2.17	0.112	1.54	0.98-2.41	0.059
Comorbidity						
No	1			1		
Yes	1.28	0.82-1.97	0.274	1.6	1.01-2.53	0.043*
Tumor stage						
T 0-2	1			1		
T 3-4	3.46	2.23-5.36	<0.0001*	3.65	2.34-5.69	<0.0001*
Lymph nodes						
Negative	1			1		
Positive	1.34	0.82-2.18	0.243	1.16	0.70-1.91	0.56
Hormonal status						
Positive	1			1		
Negative	1.62	1.02-2.57	0.042*	1.84	1.15-2.93	0.011*
Chemotherapy						
Yes	1			1		
No	0.66	0.30-1.43	0.296	1.17	0.50-2.76	0.718
HER2 status						
Negative	1			1		
Positive	1.701	1.06-2.72	0.026*	1.52	0.94-2.46	0.087
BMI (kg/m2)						
BMI < 25	1			1		
BMI ≥ 25	1.11	0.64-1.92	0.703	0.77	0.43-1.39	0.391
Age						
≥ 50	1			1		
< 50	1.41	0.81-2.45	0.218	1.49	0.85-2.61	0.164
Comorbidity						
No	1			1		
Yes	1.05	0.60-1.85	0.861	1.37	0.76-2.48	0.294
Tumor stage						
T 0-2	1			1		
T 3-4	4.46	2.55-7.82	<0.0001*	4.45	2.53-7.83	<0.0001*
Lymph nodes						
Negative	1			1		
Positive	1.58	0.83-3.01	0.167	1.23	0.63-2.41	0.538
Hormonal status						
Positive	1			1		
Negative	2.22	1.26-3.91	0.006*	2.11	1.16-3.87	0.015*
Chemotherapy						
Yes	1			1		
No	0.45	0.14-1.45	0.181	1.04	0.29-3.74	1.039
HER2 status						
Negative	1			1		
Positive	2.48	1.38-4.46	0.002*	1.98	1.07-3.65	0.029*

† HR, Hazard ratio; ‡CI, Confidence interval; * *P*-value < 0.05 statistical significance

**Table 3.** Complication of Systemic Treatment

		Total	BMI (kg/m^2^)		*P*-value
		(N = 352)	< 25	≥ 25	
			N=185	N=167	
Complication	No	187 (53.1)	89 (48.1)	98 (58.7)	0.047*
Chemotherapy	Yes	165 (46.9)	96 (51.9)	69 (41.3)	
Neutropenia	Grade 2	5 (1.4)	2 (1.1)	3 (1.8)	< 0.001*
	Grade 3	5 (1.4)	3 (1.6)	2 (1.2)	
	Grade 4	123 (34.9)	81 (43.8)	42 (25.1)	
Febrile neutropenia	No	319 (90.6)	162 (87.6)	157 (94)	0.038*
	Yes	33 (9.4)	23 (12.4)	10 (6)	
Cardiomyopathy	No	349 (99.1)	184 (99.5)	165 (98.8)	0.503
	Yes	3 (0.9)	1 (0.5)	2 (1.2)	
Dose reduction	No	190 (54)	91 (49.2)	99 (59.3)	0.058
	Yes	162 (46)	94 (50.8)	68 (40.7)	
	1st Cycle	8	0	8	
	Other cycle	154	94	60	
Complication	No	283 (92.8)	148 (91.9)	135 (93.8)	0.539
Hormonal treatment					
	Yes	22 (7.2)	13 (8.1)	9 (6.2)	
	VTE	2	2	0	
	Hepatitis	8	4	4	
	Osteoporosis	11	7	4	
	Endometrial cancer	2	1	1	

VTE, Venous thromboembolism; *p-value < 0.05 statistical significance

## Discussion

This is the first study performed in Thai patients to investigate the association between obesity defined as BMI ≥ 25 kg/m^2^ and outcome of operable breast cancer. We found that obesity had no adverse prognostic impact association on both DFS and OS in Thai breast cancer patients.

In this study, we retrospectively explored the data of operable breast cancer patients treated at Siriraj Hospital from January 2004 to December 2011, categorized by BMI into 2 groups: obese group which defined BMI ≥ 25 kg/m^2^ and non-obese group with BMI < 25 kg/m^2^. BMI cut-off points of 25 kg/m^2^ for determining obesity in Asian populations was chosen as classification proposed by the Western Pacific Region of WHO, the International Association for the Study of Obesity and the International Obesity Task Force (WHO/IASO/IOTF, 2000; Anuurad, et al., 2003). Evidence has suggested that Asians have higher body fat deposit at a lower BMI than Caucasians (WHO/IASO/IOTF, 2000). Obesity was literally reported to be associated with multiple comorbidities (Goodwin and Stambolic, 2015; Berrigan et al., 2016; Global BMI Mortality Collaboration, 2016). Obese patients in this study had significantly higher number of comorbidities including type 2 DM and hypertension, the finding confirmed that using BMI cut-off points of 25 kg/m^2^ for defining obesity is at least justified in Thai population.

Our study result was consistent with the results of many other reports showing that obese breast cancer patients were more likely to be older, postmenopausal and presented with larger tumor size and greater numbers of lymph node involvement (de Azambuja et al., 2010; Ewertz et al., 2011; Pajares et al., 2013; Ladoire et al., 2014; Widschwendter et al., 2015). The association between obesity and advanced stage may be due to a delay in diagnosis, since obese women have larger breasts which tumor are more difficult to be palpated. Another reason could be from high levels of estrone and estradiol that promote growth of tumors in obese women (Cui et al., 2002). There were no significant differences in tumor grade, hormone receptor, HER2 status, and type of surgery between obese and non-obese patients. Despite more advanced tumor stage found in obese patients, there was significant inverse association between BMI and receipt of adjuvant chemotherapy. Older age and comorbidity might be associated with a lower likelihood of receipt of chemotherapy.

The present study, performed in the tertiary-care hospital in Thailand where patients being treated with standard adjuvant chemotherapy and hormonal therapy, found that obesity defined as BMI ≥ 25 kg/m^2^ had no prognostic impact on both DFS and OS in operable breast cancer patients. A similar result from a French study reported that obesity (BMI ≥ 30 kg/m^2^) had no impact on breast cancer prognosis when modern adjuvant chemotherapy was delivered (Ladoire et al., 2014). However, several studies from Belgium (de Azambuja et al., 2010), Denmark (Ewertz et al., 2011), Japan (Kawai et al., 2016), including the most recent systematic review (Chan et al., 2014) suggested that obesity was associated with poorer outcome in breast cancer patients. All of these studies categorized patients with BMI ≥ 30 kg/m^2^ as obese. In addition, the exact association between obesity and breast cancer prognosis with respect to the level of obesity at which there is an increased risk of breast cancer recurrence has been assessed. Pajares (2013) found that patients with BMI ≥ 35 kg/m^2^ had a significantly increased risk of recurrence and mortality compared to BMI < 25 kg/m^2^, but patients with BMI 30-35 kg/m^2^ had similar prognosis as the reference group. Widschwendter (2015) reported that severe obesity (BMI ≥ 40 kg/m^2^) had worse DFS and OS. Therefore, we performed additional analysis using BMI ≥ 30 mg/m^2^ as obese, the survival plot tended to be separate between the groups with a lower 5-year DFS and OS in the obese group; nonetheless, the difference was not statistically significant. The low number of severe obesity (mean BMI = 28.6 ± 3.3 kg/m^2^) in our study may contribute to limited statistical power to prove the negative effect of severe obesity on breast cancer outcome.

In this study we found that obese patients experienced significantly less toxicity from chemotherapy with respect to grade 4 neutropenia (25% versus 44%, p < 0.001) and febrile neutropenia (6% versus 12%, p = 0.038) when compared with non-obese patients. This is in consistent with the result from a systematic review which observed that obese patients tolerated chemotherapy better than lean patients (Carroll et al., 2012). The findings were not explained by dose reduction of chemotherapy, since there was no significant difference in rate of dose reduction between the two groups. On the contrary, the obese group had less dose reduction than the non-obese patients (41% versus 51%, p = 0.058). There may be some form of neutropenia-protective mechanism in obese women such as increased chemotherapy clearance due to up-regulation of cytochrome P450 genes secondary to diets that are high in fats or carbohydrates (Murray, 2006; Carroll et al., 2014). 

The report from the International Breast Cancer Study Group (IBCSG) (Colleoni et al., 2005) showed that obese patients were significantly more likely to receive a lower chemotherapy dose for the first course than were those with normal or intermediate BMI. This translated into a significantly worse outcome particularly for the ER-negative cohort. In our study only 4.8% (8 out of 167 patients) in the obese group received their chemotherapy dose capped at an arbitrary body surface area, which was lower when compared to 15.8% reported by Caroll (2014) and 20.5% by Lote (2016). Furthermore, there was no significant in the rate of dosage reduction between the obese and non-obese group. This factor may partly explain the non-significant differences in the outcomes between the obese and non-obese group in our study. According to the American Society of Clinical Oncology (ASCO) issued guideline, full weight-based dosing is recommended in obese cancer patients (Griggs et al., 2012). There is no evidence that short- or long-term toxicity was increased among obese patients receiving full weight-based chemotherapy doses and patients with a high BMI had higher leukocyte nadirs than lean patients (Poikonen et al., 2001).

This is the first study that reported the effect of obesity defined as BMI ≥ 25 kg/m^2 ^on breast cancer outcome in Thai patients, although obesity was not found to be associated with prognosis. The data was collected from a single institution where breast cancer patients were treated in similar fashion including standard adjuvant chemotherapy with anthracycline- and taxane-based regimens. Our study did have some limitations, namely small numbers of patients when compared with previous studies that involved patients in large randomized controlled trials or population-based observational studies; the length of follow-up should be considered, since we cannot exclude the possibility that a prognostic effect of obesity may become evident after a longer follow-up time. Future study should be performed using database in cancer registry to determine the prognostic impact of obesity on breast cancer outcomes in Thai population. Meanwhile, we recommended that our patients be encouraged to maintain a healthy weight for at least general health benefits.

Obesity defined as BMI ≥ 25 kg/m^2^ did not affect outcomes of operable breast cancer in Thai patients, although obese patients presented with larger tumors and increased numbers of lymph node metastasis. Compared with non-obese patients, obese patients tended to better tolerate chemotherapy with less severe neutropenia.
